# Supplementary material for: Amyloid Beta-Mediated Changes in Synaptic Function and Spine Number of Neocortical Neurons Depend on NMDA Receptors
Source: Int J Mol Sci. 2021 Jun 11;22(12):6298. doi: 10.3390/ijms22126298 (PMC8231237; doi:10.3390/ijms22126298)
Supplement: Supplementary file 1 [file ijms-22-06298-s001.zip › ijms-1228882-supplementary.pdf]

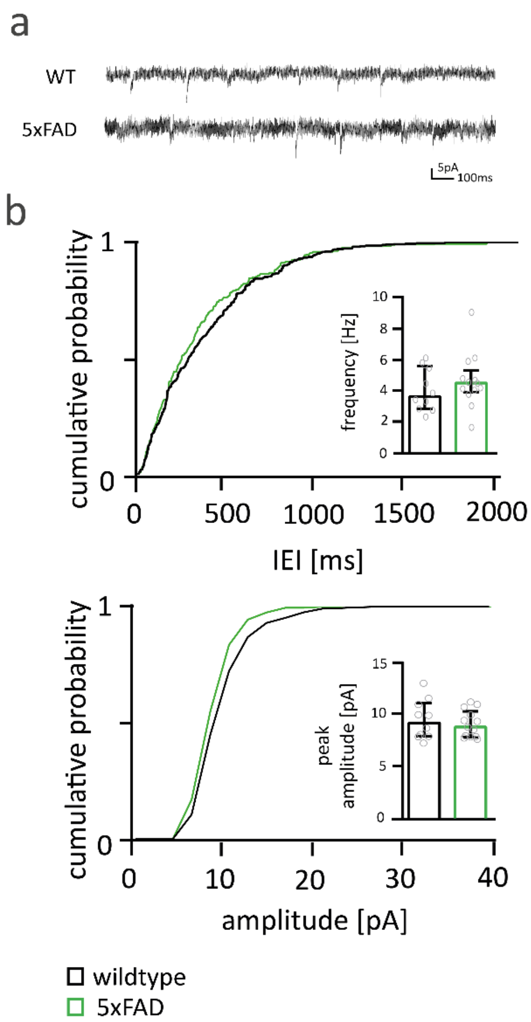


Suppl. Figure S1: mEPSCs are not different in somatosensory cortex pyramidal cells of one year old 5xFAD and WT mice. (a) Sample traces of mEPSCs recordings of pyramidal neurons of the somatosensory cortex from WT and 5xFAD mice. (b) Cumulative distribution plot of the mEPSC inter-event-intervals (IEI) and bar graph of the median mEPSC frequency of pyramidal neurons from WT (n = 10; black) and 5xFAD (n = 13; green) mice (upper part). Cumulative distribution plot of the mEPSC peak amplitudes and bar graph of the median peak amplitudes of pyramidal neurons from WT and 5xFAD neurons (lower part). Grey dots depict single data points.
